# Three-Stage Interpolation Method for Demosaicking Monochrome Polarization DoFP Images

**DOI:** 10.3390/s24103018

**Published:** 2024-05-10

**Authors:** Luping Liu, Xin Li, Jianmin Yang, Xinliang Tian, Lei Liu

**Affiliations:** 1 State Key Laboratory of Ocean Engineering, Shanghai Jiao Tong University, Shanghai 200240, China; liuluping@sjtu.edu.cn (L.L.);; 2 SJTU Yazhou Bay Institute of Deep Sea Technology, Sanya 572000, China

**Keywords:** polarized imaging, DoFP technique, MPFA demosaicking, polarization image sensor, residual interpolation

## Abstract

The emergence of polarization image sensors presents both opportunities and challenges for real-time full-polarization reconstruction in scene imaging. This paper presents an innovative three-stage interpolation method specifically tailored for monochrome polarization image demosaicking, emphasizing both precision and processing speed. The method introduces a novel linear interpolation model based on polarization channel difference priors in the initial two stages. To enhance results through bidirectional interpolation, a continuous adaptive edge detection method based on variance differences is employed for weighted averaging. In the third stage, a total intensity map, derived from the previous two stages, is integrated into a residual interpolation process, thereby further elevating estimation precision. The proposed method undergoes validation using publicly available advanced datasets, showcasing superior performance in both global parameter evaluations and local visual details when compared with existing state-of-the-art techniques.

## 1. Introduction

As optical imaging and its applications have been rapidly evolving, the study of light’s polarization properties emerges as an indispensable facet, providing higher-dimensional insights into the orientation of light wave oscillations. Th polarization of light contains rich information to facilitate a more profound comprehension and interpretation of the physical world. For example, some of the species in nature exhibit acute responsiveness to polarization cues and utilize polarized light for positioning and detection [1].

Notwithstanding the considerable potential of polarized imaging, it remains beyond the perceptual scope of the human eye, necessitating the deployment of specialized polarized imaging apparatus. Recent years have witnessed the rapid proliferation of polarized imaging and measurement technologies across diverse academic disciplines and practical applications, including image recovery [2,3], surface inspection [4], and 3D shape reconstruction [5].

**Figure 1:**
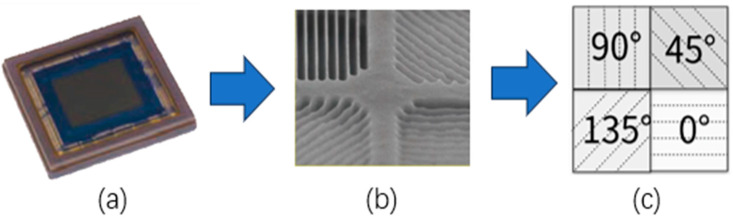
Monochrome polarizing filter array of an industrial image sensor. (**a**) Sony polarization image sensor IMX250MYR-C. (**b**) Macro image of the four directional polarizers [9]. (**c**) MPFA layout of four polarization angles.

The acquisition of polarization information from target images typically entails the capture of a set of in-site images employing polarizers placed at different angular orientations in front of the camera [6]. This enables the computational assessment and analysis of polarization attributes at the level of individual pixels within the acquired images. However, such a multi-shot approach is unsuitable for dynamic scenes and video acquisition. Recently, a novel category of polarization image sensors using the division of focal plane (DoFP) technique emerged as a pioneering sensing solution [7]. Analogous to conventional color cameras employing color filter arrays (CFAs) [8], these sensors integrate micro-polarizer arrays into the pixels of focal plane array sensors. Such polarization filter arrays (PFAs) allow for the formation of polarizers at different angles on the chip during the semiconductor-manufacturing process, enabling highly precise alignment with the pixels. The typical monochrome polarizing filter array (MPFA) used in DoFP polarization cameras consists of a 2 × 2 period of four polarizers with angles set at 0, 45, 90, and 135 degrees, as shown in Figure 1. Additionally, the color polarization filter array (CPFA) combines the principles of color imaging with polarization imaging, and features a fundamental 4 × 4 periodic structure with unique color and polarization orientation at each position.

The emergence of DoFP polarization cameras provides better opportunities for capturing polarization information, but it also presents new challenges that need to be addressed for effective full-polarization reconstruction. Image demosaicking, also referred to as mosaic interpolation, is a crucial process in digital imaging with CFAs and also PFAs. In the case of MPFAs, where each pixel location holds only one polarization orientation, retrieving data of the other polarization channels requires interpolation or reconstruction for accurate estimation. MPFA demosaicking based on interpolation-based methods is also an integral component of CPFA interpolation-based demosaicking. This process is oriented towards the completion of absent polarization information at each pixel location by extrapolating values of the other three polarization orientations from the pixel’s neighborhood.

Compared to CFA demosaicking [10,11], that with MPFAs inherently presents greater complexity due to the increased number of channel information within an array and the challenges in addressing intricate polarization characteristics. The effective polarization estimation and management of inter-channel correlations become imperative in this context. In tackling the demosaicking challenge in monochrome polarization images, some conventional interpolation methods employed in CFA demosaicking, including bilinear interpolation, bicubic interpolation, and cubic spline interpolation, have been adapted for MPFA demosaicking [12]. However, these methods primarily rely on spatial correlations and overlook inter-channel correlations. Given the disparities in array types, many well-structured CFA demosaicking algorithms may also not achieve optimal results, owing to inherent distinctions in color and polarization mechanisms [13]. Zhang et al. [14] proposed an interpolation method that enhances the demosaicking quality of DoFP polarization images using intensity correlations. They performed dual bicubic interpolation with edge detection for both oblique and vertical pixels, leading to improved results. Recognizing that high-frequency information has lower polarization direction differences, Li et al. [15] proposed a polarization differential domain interpolation method using Newton polynomial interpolation. Morimatsu et al. [16] presented a method using intensity-guided edge-aware residual interpolation based on channel differences, demonstrating notable improvements despite the requirement for complex computations. In a recent development, Wu et al. [17] introduced a simple yet effective polarization image demosaicking technique, leveraging prior information about polarization channel differences to process MPFA demosaicking. While Xin et al. [18] made further enhancements, neither one nor the other of these methods yielded significantly superior results.

Numerous learning-based methods have also been proposed and applied to the PFA demosaicking in recent years, and some have achieved commendable results. For MPFA demosaicking specifically, there exist dictionary-learning-based [19,20,21,22] and deep-learning-based methods [23,24,25,26], but their efficacy is highly contingent on the datasets employed during training and the demand a high computational costs and runtimes. These limitations underscore the impracticality of their actual applications in image sensors and emphasize the crucial need to further investigate intensity-based interpolation methods.

In this paper, we present a fast and highly effective demosaicking model comprising a three-stage interpolation procedure designed to address this challenging issue. Our model achieves superior results compared with state-of-the-art methods. The first two stages introduce a novel linear interpolation method tailored for MPFAs, incorporating customized weights for neighboring pixel values. For the first stage, we separately interpolate along the 45-degree and 135-degree diagonal directions to recover the missing polarization information that is perpendicular to the polarizer angle formed on the chip. A continuous adaptive edge detection method that relies on the variance difference between these two diagonal directions is then utilized to perform weighted averaging for this bidirectional interpolation. For the second stage, we apply the same method twice in both horizontal and vertical directions, thereby acquiring pixel values in the remaining two absent directions for each pixel location. The complete polarization information for each pixel position is obtained hereto, but we choose to refine the results through a residual interpolation step in the third stage. Instead of using the residual value between two different polarization orientations, we utilize the total intensity value calculated from the results obtained in the previous stages.

Our proposed method has undergone validation on publicly available datasets, demonstrating its superior performance in global evaluation and local details when compared with existing intensity-based interpolation methods. Additionally, it offers relatively fast computational times and holds the potential for further enhancement through parallel acceleration.

## 2. Methods

### 2.1. Polarization Channel Difference Interpolation

Most CFA interpolation methods are based on color channel difference interpolation [27,28]. The same holds true for PFA interpolation, as many studies have discussed the difference interpolation between polarization channels [15,17,18]. After various tests and verification, a relatively reliable assumption is made: high-frequency energy is reduced in the polarization channel difference domain [17]. By using the channel difference for interpolation, demosaicking accuracy can be improved compared with that of independent interpolation for each polarization channel.

**Figure 2:**
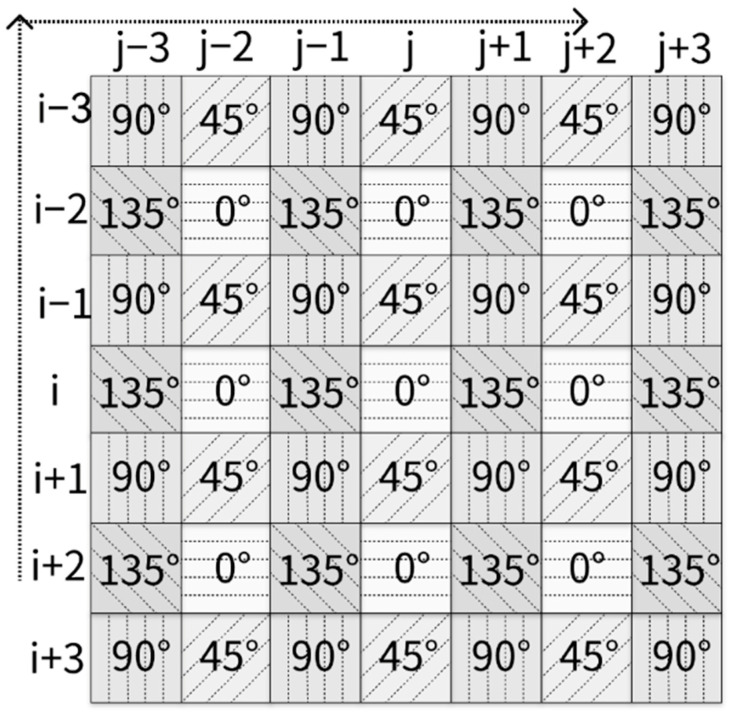
A classic 7 × 7 MPFA pattern with rectangular coordinates for algorithm representation. For example, $\left(i,j\right)$ is the location of the center pixel in the pattern.

The purpose of basic linear polarization channel difference interpolation is to obtain the average of the polarization channel differences on both sides of the pixel. Figure 2 is a 7 × 7 pixel array arranged in an MPFA pattern. Each angle marked in a small square represents the polarization orientation at a specific pixel position within the MPFA. In Figure 2, $I_{0}\left(i,j\right)$ is the available information in the central position, while $I_{d}\left(i,j\right)$, $d\in \left\{45,90,135\right\}$, denotes the missing values that require interpolation. For illustration, only horizontal interpolation is performed in this section, and the polarization information of orientation 135° can be estimated in position $\left(i,j\right)$, as shown below:

(1) $${\hat{I}}_{135}^{\cdots }\left(i,j\right)-I_{0}\left(i,j\right)=\frac{I_{135}\left(i,j-1\right)-{\tilde{I}}_{0}\left(i,j-1\right)}{2}+\frac{I_{135}\left(i,j+1\right)-{\tilde{I}}_{0}\left(i,j+1\right)}{2}$$

To avoid confusion with polarization orientations, “⋯” is used in this paper to represent the horizontal interpolating direction. We use “~” to indicate the unknown value that needs to be temporarily represented and does not participate in the actual calculation process. “^” is used to represent the estimated result obtained in each interpolation stage. In Equation (1), ${\hat{I}}_{135}^{\cdots }\left(i,j\right)$ represents the value estimated through horizontal interpolation. All remaining terms except for ${\tilde{I}}_{0}\left(i,j-1\right)$ and ${\tilde{I}}_{0}\left(i,j+1\right)$ are known in the original MPFA image. The challenge then lies in estimating these two unknown values. Most methods simply treat these two terms as averages of their respective left and right neighbors [13].

(2) $${\tilde{I}}_{0}\left(i,j\pm 1\right)=\frac{I_{0}\left(i,j\right)+I_{0}\left(i,j\pm 2\right)}{2}$$

After rearranging Equations (1) and (2), we obtain the following:

(3) $${\hat{I}}_{135}^{\cdots }\left(i,j\right)=\frac{I_{135}\left(i,j-1\right)+I_{135}\left(i,j+1\right)}{2}-\frac{I_{0}\left(i,j-2\right)+I_{0}\left(i,j+2\right)-2I_{0}\left(i,j\right)}{4}$$

where the first term is the first-order mean, and the second term, $\frac{I_{0}\left(i,j-2\right)+I_{0}\left(i,j+2\right)-2I_{0}\left(i,j\right)}{4}$, which could be written as $\partial^{2}{\hat{I}}_{135}^{\cdots }\left(i,j\right)$, represents the second-order gradient.

While this method is effective, it may not provide sufficient precision for MPFA interpolation at a higher level. If we repeatedly estimate ${\tilde{I}}_{0}\left(i,j-1\right)$ and ${\tilde{I}}_{0}\left(i,j+1\right)$ using the same method as that applied to ${\hat{I}}_{135}^{\cdots }\left(i,j\right)$ in Equation (1), it will contain a relatively high proportion of $I_{135}\left(i,j-1\right)$ and $I_{135}\left(i,j+1\right)$. This would be consequently subtracted by the first-order mean and potentially impact the primary interpolation results.

Therefore, we propose a compromise solution in which we replace the interpolation of the polarization channel difference, ${\tilde{I}}_{0}\left(i,j\pm 1\right)-I_{135}\left(i,j\pm 1\right)$, with a new approach: ${\tilde{I}}_{0}\left(i,j\pm 1\right)-\frac{1}{2}I_{135}\left(i,j\pm 1\right)$. Then, Equation (2) is replaced by the following:

(4) $${\tilde{I}}_{0}\left(i,j\pm 1\right)-\frac{1}{2}I_{135}\left(i,j\pm 1\right)=\frac{I_{0}\left(i,j\right)-{\frac{1}{2}\tilde{I}}_{135}\left(i,j\right)}{2}+\frac{I_{0}\left(i,j\pm 2\right)-{\frac{1}{2}\tilde{I}}_{135}\left(i,j\pm 2\right)}{2}$$

where ${\tilde{I}}_{135}\left(i,j\right)$ and ${\tilde{I}}_{135}\left(i,j\pm 2\right)$ can be obtained by averaging their neighbors, as in Equation (2). After simplifying Equations (1) and (4), we arrive at the final interpolation procedure in this stage:

(5) $${\hat{I}}_{135}^{\cdots }\left(i,j\right)=\frac{7I_{135}\left(i,j-1\right)+7I_{135}\left(i,j+1\right)+I_{135}\left(i,j-3\right)+I_{135}\left(i,j+3\right)}{16}-\partial^{2}{\hat{I}}_{135}^{\cdots }\left(i,j\right)$$

### 2.2. Interpolation Sequence and Edge Detection

Section 2.1 exclusively focuses on clarifying linear interpolation in a single direction. However, in many cases, the values within an image can exhibit significant variations, especially near edges. When solely relying on one-directional interpolation for missing values, the resulting outcome may lack reliability. Consequently, there is a need for multi-directional interpolation. In actual practice, bidirectional interpolation is commonly employed, while edge detection is then highly required for directional weighting.

In the process of CFA demosaicking, it is a common practice to prioritize the interpolation of the green channel, both horizontally and vertically, due to it having double the number of pixels compared with other color channels. However, as for MPFA interpolation, polarization information is uniformly distributed in channels across the four directions on the chip, as shown in Figure 1. Bidirectional interpolation for missing polarization information poses a more challenging task.

**Figure 3:**
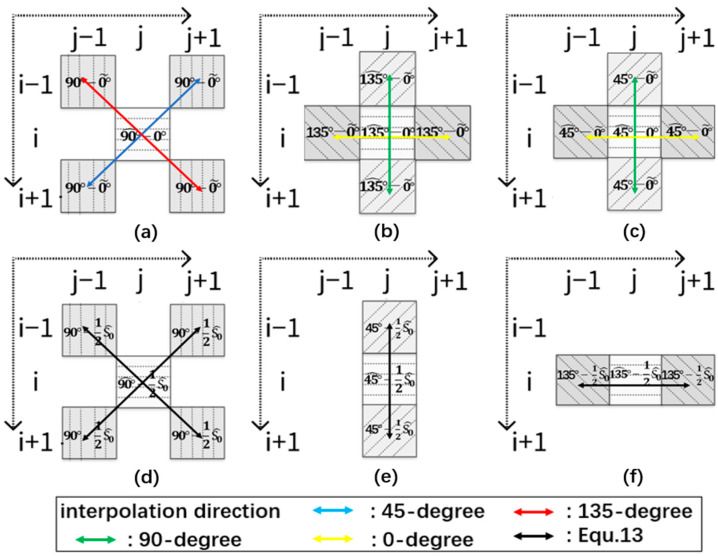
Illustration of interpolation procedure at coordinate $\left(i,j\right)$ using our three-stage MPFA demosaicking method. The degrees within the grid represent the intensity values of the corresponding polarization orientation at each location. (**a**) The first stage of obtaining ${\hat{I}}_{90}\left(i,j\right)$. (**b**,**c**) The second stage of obtaining ${\hat{I}}_{135}\left(i,j\right)$ and ${\hat{I}}_{45}\left(i,j\right)$, respectively. (**d**–**f**) The third stage of obtaining the final estimation of ${\hat{I}}_{90}\left(i,j\right)$, ${\hat{I}}_{45}\left(i,j\right)$, and ${\hat{I}}_{135}\left(i,j\right)$.

In our initial stage, we employ diagonal interpolations along the 45-degree and 135-degree directions as a deliberate choice, instead of common interpolation starting from horizontal and vertical directions. This approach allows us to simultaneously estimate information from two perpendicular directions and thus minimizes interference stemming from incorrect directional interpolation near image edges. We can then gather polarization information perpendicular to the polarization orientation of each pixel’s polarizer on the MPFA, as illustrated in Figure 3a.

Similarly, we use “⋰” and “⋱” to denote the 45-degree and 135-degree interpolation directions, respectively. ${\hat{I}}_{90}\left(i,j\right)$ in bidirectional interpolation can then be expressed by the following equation:

(6) $${\hat{I}}_{90}\left(i,j\right)=\omega_{90}^{⋰}\left(i,j\right){\hat{I}}_{90}^{⋰}\left(i,j\right)+\omega_{90}^{\ddots }\left(i,j\right){\hat{I}}_{90}^{\ddots }(i,j)$$

where ${\hat{I}}_{90}^{⋰}\left(i,j\right)$ and ${\hat{I}}_{90}^{\ddots }(i,j)$ are the results of interpolation in the respective directions. While $\omega_{90}^{⋰}\left(i,j\right)$ and $\omega_{90}^{\ddots }\left(i,j\right)$ represent the coefficients of directional weights assigned to their corresponding directions, $\omega_{90}^{⋰}\left(i,j\right)+\omega_{90}^{\ddots }\left(i,j\right)=1$ can be obtained. Both weights are determined based on the rate of change of pixel values in the respective directions within a specific distance. In many studies [13,14,27], the rate of change, taking the position $\left(i,j\right)$ along the 45-degree interpolation direction as an example, is typically defined as follows:

(7) $$\nu_{90}^{⋰}\left(i,j\right)=\left|I_{0}\left(i-1,j+1\right)-I_{0}\left(i+1,j-1\right)\right|+4*\left|\partial^{2}{\hat{I}}_{90}^{⋰}\left(i,j\right)\right|$$

When changes along a certain direction are more rapid than those along the perpendicular direction, they imply the potential presence of an edge at the corresponding pixel location. In such cases, it is advisable to assign a smaller coefficient to that direction, given the uneven variation across the edge along that direction. Many edge detection methods adopt a similar coefficient assignment strategy: if one direction undergoes more significant changes than the other, the coefficient for that direction is set to 0, and in the converse scenario, it is set to 1. When the changes in both directions are equal, the coefficients for both directions are set to 0.5 [8,27]. However, this common approach may result in discontinuities across the image and improper estimated values due to the rare occurrence of $\nu_{90}^{⋰}\left(i,j\right)=\nu_{90}^{\ddots }\left(i,j\right)$. This leads to a situation where unidirectional interpolation is predominantly employed in practical applications instead of the expected bidirectional interpolation.

An enhanced edge detection method incorporates the utilization of gradients in both directions and computes coefficients in accordance with the following formulation:

(8) $$\omega_{90}^{⋰}\left(i,j\right)=\frac{\nu_{90}^{\ddots }\left(i,j\right)}{\nu_{90}^{⋰}\left(i,j\right)+\nu_{90}^{\ddots }\left(i,j\right)+\varepsilon }$$

where ε is a small coefficient introduced to avoid division by zero. This method is more effective and robust. However, in cases where $\nu_{90}^{⋰}\left(i,j\right)$ and $\nu_{90}^{⋰}\left(i,j\right)$ manifest substantial disparities, the smaller weight still contributes as a non-negligible part. This often necessitates the additional establishment of complex boundary conditions for constraints [15]. These conditions are typically based on their ratio, but may lack universality for various image patterns. Moreover, when $\nu_{90}^{⋰}\left(i,j\right)$ and $\nu_{90}^{⋰}\left(i,j\right)$ are small values, the ratio between them can easily become imbalanced, leading to an approximation of unidirectional interpolation. In light of this, directional coefficients are suggested to be established based on the difference between the rates of change in this paper. To ensure that the weights change continuously with the difference, $\omega_{90}^{⋰}\left(i,j\right)$ and $\omega_{90}^{\ddots }\left(i,j\right)$ should have the same form and satisfy the following requirements:

(9) $$\omega_{90}^{⋰}\left(i,j\right){+\omega }_{90}^{\ddots }\left(i,j\right)=f\left(\nu_{90}^{⋰}\left(i,j\right)-\nu_{90}^{\ddots }\left(i,j\right)\right)+f\left(\nu_{90}^{\ddots }\left(i,j\right)-\nu_{90}^{⋰}\left(i,j\right)\right)=1$$

where f represents the custom function determined to satisfy the equation and maintain the sum of the two directional weights. Similar to the approach in [27], the following function is selected to determine the coefficients of directional weights:

(10) $$\omega_{90}^{⋰}\left(i,j\right)=\frac{1}{1+e^{k(\nu_{90}^{⋰}\left(i,j\right)-\nu_{90}^{\ddots }\left(i,j\right))}}and\;\omega_{90}^{\ddots }\left(i,j\right)=\frac{1}{1+e^{k(\nu_{90}^{\ddots }\left(i,j\right)-\nu_{90}^{⋰}\left(i,j\right))}}$$

where k is a positive number used to adjust the changes of weights. In our experiments, we set parameter k to 20 when pixel values in the MPFA image ranged from 0 to 1.

Once we acquired the polarization information of each pixel in the orthogonal direction through the first stage of interpolation, we proceed with the same method in the vertical and horizontal directions to obtain the remaining missing information for each pixel. For position $\left(i,j\right)$, the second stage aims to obtain ${\hat{I}}_{135}\left(i,j\right)$ and ${\hat{I}}_{45}\left(i,j\right)$, as shown in Figure 3b,c.

To illustrate, the estimation of ${\hat{I}}_{45}\left(i,j\right)$ through bidirectional interpolation is taken as an example, as shown in Equation (11). The vertical result, ${\hat{I}}_{45}^{\vdots }\left(i,j\right)$, is obtained through interpolation based on values directly from the original MPFA image and goes in the same direction as the green arrow does. On the other hand, ${\hat{I}}_{45}^{\cdots }\left(i,j\right)$ is derived from interpolation along the direction of the yellow arrow, utilizing the results obtained from the initial stage of our procedure to proceed with the second stage:

(11) $${\hat{I}}_{45}\left(i,j\right)=\omega_{45}^{\cdots }\left(i,j\right){\hat{I}}_{45}^{\cdots }\left(i,j\right)+\omega_{45}^{\vdots }\left(i,j\right){\hat{I}}_{45}^{\vdots }\left(i,j\right)$$

### 2.3. Third-Stage Interpolation

After completing two stages of bidirectional interpolations, as described in 2.1 and 2.2, the full polarization information was retrieved and the results were already adequate for calculating the polarization parameters, including total light intensity (${\hat{S}}_{0}$), degree of linear polarization (DOLP), and angle of linear polarization (AOLP).

However, we decided to go further to enhance estimation accuracy by employing a method similar to that of residual interpolation in CFA [11]. For each position, we computed the mean of the four polarization orientations, which accounts for half of the total intensity. For position (i,j) in Figure 2, the mean value is calculated via the following:

(12) $$\frac{1}{2}{\hat{S}}_{0}(i,j)={(I}_{0}\left(i,j\right)+{\hat{I}}_{90}\left(i,j\right)+{\hat{I}}_{45}\left(i,j\right)+{\hat{I}}_{135}\left(i,j\right))*0.25$$

**Figure 4:**
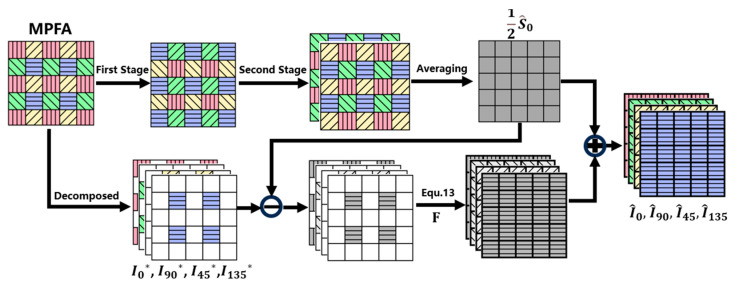
Overall process of our proposed method.

Next, we calculated the discrepancy between the mean value and the corresponding pixel value in the original MPFA image, resulting in the residual value for each pixel position. The residual map is decomposed into four images, ${I_{0}}^{*}$, ${I_{90}}^{*}$, ${I_{45}}^{*}$, and ${I_{135}}^{*}$, as illustrated in Figure 4. Each contains residuals of a different orientation, with the remaining positions filled with zeros. Subsequently, we performed linear interpolation of these four images via convolution. After incorporating the mean value, we restored the original MPFA values and updated pixel values at zero positions for these four images, as shown in the following formula:

(13) $$I_{D}=\left(I_{MFPA}^{maskD}-\frac{1}{2}{{\hat{S}}_{0}}^{maskD}\right)*F+\frac{1}{2}{\hat{S}}_{0}$$

where $I_{MFPA}^{maskD}$ is one of the decomposed images, with D∈{0,45,90,135}, and F is the filter of the convolution:

(14) $$F=\frac{1}{4}\left[\begin{array}{ccc} 1 & 2 & 1\\ 2 & 4 & 2\\ 1 & 2 & 1 \end{array}\right]$$

Taking pixel position (i,j) in Figure 2 as an example, the original value, $I_{0}\left(i,j\right)$, in MPFA remains unchanged in ${I_{0}}^{*}$, while the additional polarization values ${\hat{I}}_{90}\left(i,j\right)$, ${\hat{I}}_{45}\left(i,j\right)$, and ${\hat{I}}_{135}\left(i,j\right)$ are estimated, respectively, in ${I_{90}}^{*}$, ${I_{45}}^{*}$, and ${I_{135}}^{*}$, as illustrated in Figure 3d–f. The entire procedure of our proposed method, including the first two stages, is illustrated in Figure 4. This diagram provides a comprehensive overview of the process, and the final estimation of the complete polarization information can be obtained from MPFA.

## 3. Experiment

To evaluate the effectiveness of our proposed method, we employed a publicly available dataset [16], comprising 40 distinct scenes. Each scene consisted of 12 monochrome images, each with a size of 1024 × 768 pixels, representing different color channels and polarization orientations. Notably, this dataset was captured using a 3-CCD camera, enabling the separate recording of each color channel. Unlike images captured with common cameras, sets of four images with the same color channel and scene but taken at different polarizer angles (0°, 45°, 90°, and 135°), excluding the need for CFA demosaicking, prove to be a more suitable choice for evaluating MPFA demosaicking solutions compared with other public datasets.

**Figure 5:**
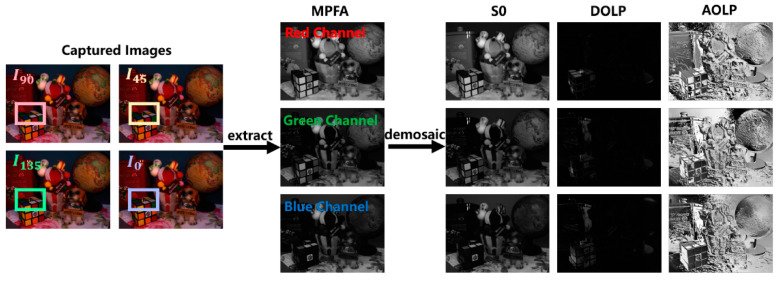
Input and output of the demosaicking algorithm.

In our study, we utilized and tested all three color channels separately for experiments. To simulate the polarization images captured by an actual monochrome DoFP camera, each set was down-sampled, creating an MPFA pattern identical to the one depicted in Figure 2. Consequently, each scene, stored in four captured color images, could generate three simulated MPFA images for testing, as illustrated in Figure 5.

Besides our proposed method, we also tested other state-of-the-art demosaicking algorithms to demonstrate our method’s superiority, including intensity correlation in polarization channel (ICPC, 2016) [14], pseudo-panchromatic image difference (PPID, 2018) [13], Newton’s polynomial interpolation and polarization difference (NPPD, 2019) [15], edge-aware residual interpolation (EARI, 2019) [16], polarization channel difference prior (PCDP, 2021) [17], and polarization difference based on edge compensation (PDEC, 2023) [18], on the extracted down-sampled images with an MPFA pattern. The codes of all these methods were downloaded from the internet and implemented in MATLAB. We slightly adjusted and standardized these codes for comparative purposes. This ensured the consistency of the input and output formats of various methods for the effective evaluation of performance during the comparison.

The full polarization information (I0, I45, I90, and I135) of each pixel position could be obtained with these methods. Polarization parameters S0, DoLP and AoLP are also computed separately using different methods for comparison. For quality assessment, we compared the recovered results with ground truth values to evaluate the performance of each method. The root mean square error (RMSE) is selected in this paper to quantify how far predictions are from the ground truth values, and a lower RMSE indicates better performance.

(15) $$RMSE=\sqrt{\frac{1}{mn}\sum_{i=0}^{m-1}\sum_{j=0}^{n-1}{{(I}_{p}^{est}(i,j)-I_{p}^{gt}(i,j))}^{2}}$$

where m and n represent the pixel dimensions of the images. In the dataset used in our study, m = 1024, and n = 768. $I_{p}^{est}$ denotes the estimated value of the compared polarization parameter, and $I_{p}^{gt}\;$is the ground truth value obtained from the captured images. In some previous studies, PSNR was regarded as a critical indicator of image quality [11,20]. However, it is essential to note that the range of PSNR varies at different scales and is represented on a logarithmic scale. It can be readily derived from RMSE, as shown in Equation (16):

(16) $$PSNR=20\times \log_{10}\left(\frac{{MAX}_{I}}{RMSE}\right)$$

where ${MAX}_{I}$ is the maximum possible pixel value of the images. During these measurements, we excluded the 12-pixel border around the images to mitigate any boundary effects related to demosaicking. Additionally, we measured the runtime for each demosaicking method.

In order to enhance the visualization and make better comparisons, the error images based on an intensity value of S0 obtained through different methods mentioned above are presented, which quantify the deviation of the estimated value from the ground truth. Considering the difficulty in comparing the DOLP results in Figure 5, we simultaneously represent the estimated values of both DOLP and AOLP within an image, following the structure of HSV color space, which is analogous to that in [16]. Specifically, we mapped the range of AOLP angles to the hue channel within the [0, π] range. Given that DOLP values tend to be relatively small, we scaled them up by a factor of 20 and mapped the resulting values to the saturation channel. The V channel was kept constant at 0.5 to maintain the image intensity at a moderate level. These visual comparisons in specific regions allowed us to depict the polarization information in a manner that made it easier to discern and compare the outcomes achieved through various methods.

## 4. Discussion

### 4.1. Overall Performance Comparison

Table 1 presents the average RMSE values of the 40 scenes in a single-color channel within the dataset for different demosaicking methods. One color channel in a particular scenario may exhibit a considerably higher intensity compared with the other two channels, leading to notable disparities in the error of estimated results during MPFA demosaicking. Consequently, comparison results obtained from a single-color channel may vary. The average RMSE of the three color channels are thus all presented, and the results of these methods reveal a nearly consistent difference in deviation among the three color channels, effectively demonstrating and distinguishing the varying performance of these methods.

Among these results, our method consistently demonstrates superior performance across most parameters, encompassing the four polarization images (I0, I45, I90, and I135),Stokes image S0, and the AOLP image. The EARI method also exhibits noteworthy efficacy among the existing methods, particularly in DOLP results, as it attains the lowest RMSE values in DOLP images within the green and blue color channels. However, it does not match the overall performance of our method across most parameters.

Moreover, it is important to note that the EARI method necessitates significantly more computational time compared with other methods, whereas our method operates nearly three times faster than EARI does under the same conditions, as shown in Table 2.

### 4.2. Visual Performance in Specific Regions

**Figure 6:**
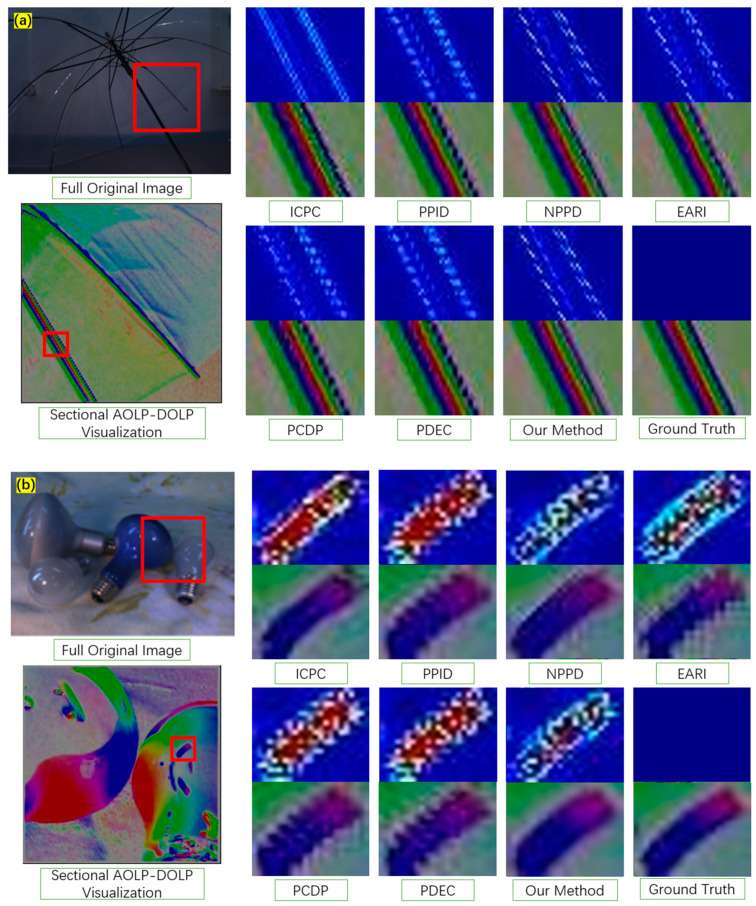
Visualization in specific regions of original data, (**a**) “Umbrella”; (**b**) “Lamp”. Comparisons in upper line: error images of S0 and lower line: AOLP-DOLP images.

In the context of images captured using MPFA cameras, it is crucial to note that regions with reflections and glass surfaces may exhibit more pronounced variations in polarization information compared with other areas. Nevertheless, these specific scenarios may not be adequately represented within the dataset. Consequently, it becomes imperative to account for these cases when assessing demosaicking algorithms. We then extracted specific 256 × 256-pixel regions from the captured images in the dataset, where particular objects such as bottles and bulbs occupy a significant portion of the region, as illustrated by the red box region from the original image in Figure 6. These were chosen to assess the algorithm’s performance in these particular scenarios.

For detailed comparison, we further selected specific local regions from the AOLP–DOLP results. The error images based on intensity are also presented, which quantify the deviation from the ground truth of S0. We use pseudo-colored images to represent the degree of deviation of each pixel location within the selected region. The vivid visual representations in these specific regions serve as a testament to the effectiveness of our approach in preserving the finer details of polarization information. The pseudo-colored images, which span from blue to red, offer a clear and intuitive means of conveying the extent of deviation in each pixel within the selected regions. Notably, the ground truth is consistently depicted in blue, allowing for the easy differentiation and comparison of outcomes achieved via various methods.

In the comparative analysis of the localized regions selected from the two scenarios, it is evident that our method outperforms others. In the S0 domain, the degree of deviation is relatively low, indicating the accuracy and precision of our approach in capturing polarization information. Within the AOLP–DOLP representation, we observe a reduced level of blurriness, further underlining the superior performance of our technique. In conclusion, the localized analysis of these specific regions not only showcases the superior performance of our method in capturing polarization information with precision but also highlights the reduced blurriness within the AOP–DOP domain. These results affirm the effectiveness and potential of our approach in applications involving MPFA cameras and situations with reflections and glass surfaces, which often present challenges in polarization imaging.

## 5. Conclusions

In summary, we presented a novel MPFA demosaicking model that demonstrated exceptional performance, surpassing current state-of-the-art techniques in various crucial aspects. Through a well-structured three-stage interpolation procedure, we achieved remarkable success in the more accurate recovery of polarization information, setting a new benchmark in the field. The validation on a public diverse dataset highlights our method’s superiority in terms of RMSE values, especially in capturing different polarization parameters including the four polarization images (I0, I45, I90, and I135), Stokes image S0, and the AOLP image. Specific region analysis further underscored the precision and accuracy of our technique, particularly in challenging scenarios involving reflections and glass surfaces. These results collectively underscore the potential and effectiveness of our approach in the realm of MPFA cameras and challenging polarization imaging applications.

Looking ahead, we are committed to exploring algorithm optimization to enhance computational efficiency. Though our approach excels in computational efficiency, operating nearly three times faster than the closest competitor, it still has high potential for further acceleration. Moreover, our work will encompass a broader range of environmental conditions and scene complexities, ensuring the robustness and versatility of our approach. This ongoing research will undoubtedly contribute to the continual evolution of polarization imaging, with far-reaching implications in domains including remote sensing, medical diagnostics, and advanced material characterization.

## Figures and Tables

**Table 1:** The average RMSE comparisons between different methods for each color channel.

Color Channel	Parameter (Scaling)	ICPC (2016)	PPID (2018)	NPPD (2019)	EARI (2021)	PCDP (2021)	PDEC (2023)	TSI (Our)
Red Channel	I90(E-3)	7.99	5.59	4.82	4.77	5.19	5.70	**4.43**
	I45(E-3)	9.20	7.30	7.13	6.77	6.89	6.90	**6.53**
	I135(E-3)	9.12	7.34	7.29	6.95	7.00	7.01	**6.83**
	I0(E-3)	8.36	6.02	5.18	5.14	5.59	6.07	**4.84**
	S0(E-3)	6.00	4.45	3.79	3.91	4.15	4.34	**3.74**
	DoLP(E-2)	1.80	1.47	1.74	1.47	1.51	**1.46**	1.54
	AoLP(E-1)	2.25	2.06	2.04	2.02	2.04	2.08	**1.98**
Green Channel	I90(E-3)	7.56	5.35	4.57	4.56	4.99	5.47	**4.21**
	I45(E-3)	8.76	7.04	6.89	6.55	6.70	6.68	**6.32**
	I135(E-3)	8.73	7.10	7.05	6.73	6.81	6.81	**6.59**
	I0(E-3)	7.85	5.69	4.91	4.89	5.33	5.78	**4.59**
	S0(E-3)	5.67	4.26	3.64	3.75	4.00	4.17	**3.57**
	DoLP(E-2)	2.09	1.72	1.96	**1.66**	1.74	1.70	1.77
	AoLP(E-1)	2.14	1.95	1.94	1.91	1.93	1.97	**1.88**
Blue Channel	I90(E-3)	8.02	5.75	4.89	4.97	5.37	5.86	**4.53**
	I45(E-3)	9.26	7.48	7.31	6.95	7.12	7.12	**6.70**
	I135(E-3)	9.15	7.47	7.41	7.08	7.18	7.17	**6.91**
	I0(E-3)	8.21	5.99	5.18	5.25	5.64	6.08	**4.87**
	S0(E-3)	5.96	4.51	3.84	4.00	4.25	4.41	**3.78**
	DoLP(E-2)	2.05	1.69	1.93	**1.62**	1.71	1.69	1.74
	AoLP(E-1)	2.40	2.23	2.23	2.20	2.22	2.25	**2.18**

**Table 2:** Running time comparisons between different methods for a single MPFA demosaicking process.

	ICPC (2016)	PPID (2018)	NPPD (2019)	EARI (2021)	PCDP (2021)	PDEC (2023)	TSI (Our)
Time (s)	0.167	0.150	0.364	0.987	0.628	0.617	0.342

## Data Availability

Data will be made available on request.
